# Determination of melatonin content in traditional Thai herbal remedies used as sleeping aids

**DOI:** 10.1186/2008-2231-22-6

**Published:** 2014-01-06

**Authors:** Tanit Padumanonda, Jeffrey Johns, Autcharaporn Sangkasat, Suppachai Tiyaworanant

**Affiliations:** 1 Division of Pharmacognosy and Toxicology, Faculty of Pharmaceutical Sciences, Khon Kaen University, Khon Kaen 40002, Thailand; 2 Office of Academic Affairs and the Melatonin Research Group, Faculty of Pharmaceutical Sciences, Khon Kaen University, Khon Kaen 40002, Thailand

**Keywords:** Melatonin, Sleep aids, Thai herb, Solid phase extraction, HPLC

## Abstract

**Background:**

Melatonin content was screened in leaves of seven edible herbs used as sleeping aids in Thai traditional medicine. These plants are *Piper nigrum L, Sesbania glandiflora* (L.) Desv., *Sesbania sesban* (L.) Merr., *Senna tora* (L.) Roxb., *Moringa oleifera* Lam., *Momordica charantia* L. and *Baccaurea ramiflora* Lour. Dried leaves were extracted by sonication in methanol for six hours at room temperature, and then melatonin was purified by C18 solid phase extraction (SPE). Melatonin was then quantified by a validated RP-C18 HPLC method with fluorescent detection.

**Findings:**

Melatonin contents in extracts of *B. ramiflora,* *S. glandiflora,* *M. charantia,* *S. tora* and *S. sesban* were 43.2, 26.3, 21.4, 10.5 and 8.7 ng/g of dry sample weight, respectively. The highest melatonin content was from *P. nigrum* extract (1092.7 ng/g of dry sample weight). Melatonin was not detected in the extract of *M. oleifera*. Melatonin identification was confirmed by ELISA.

**Conclusions:**

Melatonin was found in six of the seven herbs in the traditional Thai sleeping recipe. One of these, *P. nigrum,* exhibited an encouragingly high amount of melatonin.

## Findings

Short communication.

### Introduction

Melatonin (N-acetyl-5-methoxytryptamine) is a neuroendocrine hormone produced primarily by the pineal gland in the brain from the amino acid tryptophan, stimulated by darkness and suppressed by light. Melatonin is involved in circadian rhythm and regulation of diverse body functions, including sleep [1].

Melatonin seems to be almost universal, having been found in every vertebrate so far screened, in unicellular organisms, and in leaves, flowers, fruits and seeds of many plants [2]. Synthetic melatonin is widely used as in prevention of migraine and treatment of insomnia [3], and for jet lag [4] in many countries. In most of these, however, melatonin is only available as an imported drug indicated for the short-term treatment of primary insomnia characterized by poor quality of sleep. Therefore there is increasing interest in finding natural sources of melatonin, particularly as a sleeping aid [5].

**Figure 1:**
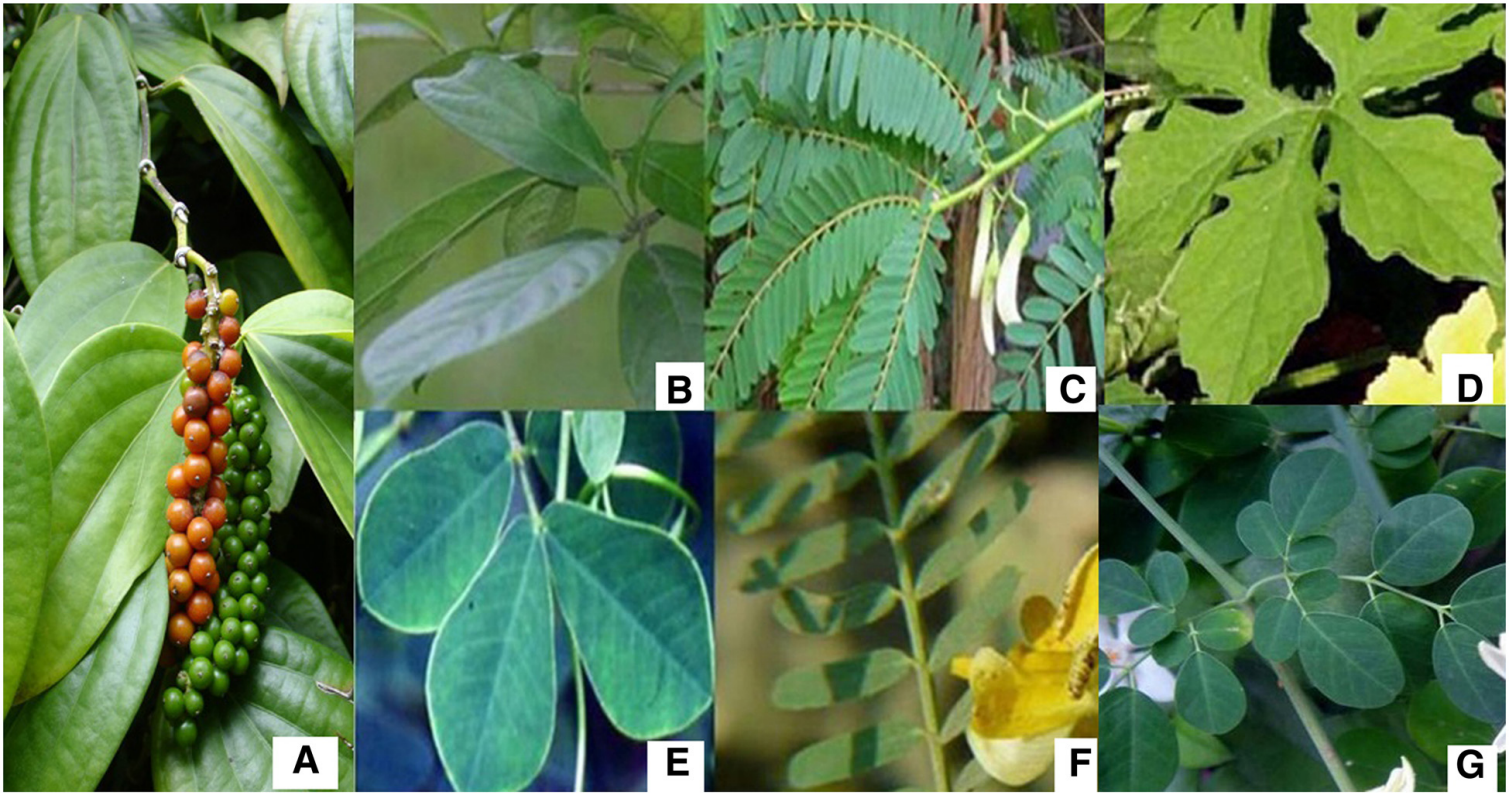
**Seven plants used as sleeping aids in Thai traditional medicine.** **(A)** *Piper nigrum* L. **(B)** *Baccaurea ramiflora* Lour., **(C)** *Sesbania glandiflora* (L.) Desv. **(D)** *Momordica charantia* L. **(E)** *Sesbania sesban* (L.) Merr. **(F)** *Senna tora* (L.) Roxb. **(G)** *Moringa oleifera* Lam.

In Thai traditional medicine, there are seven edible herbs recommend as sleeping aids. These herbs are *Piper nigrum* L, *Sesbania glandiflora* (L.) Desv., *Sesbania sesban* (L.) Merr., *Senna tora* (L.) Roxb, *Moringa oleifera* Lam., *Momordica charantia* L. and *Baccaurea ramiflora* Lour (Figure 1). Formulations of these plants are included in the Thai medical text book called Tumra Paetsart Sonkrau Chabub Anurak – Textbooks of Thai Traditional Medicine [6], as sleeping aids. This textbook is a compilation of Thai Traditional Medicine and Ayurvedh Vidhayalai College elaborating the content with scientific explanations and using contemporary language for easy understanding by lay people and students. The textbook indicates that consumption of these seven cooked mature leaves with rice is believed to increase "blood tonic" and induce sleep.

A literature review of the seven plants concluded the following. *P*. *nigrum*; or Black Pepper (Family Piperaceae) is a climbing vine native to Southern India and Sri Lanka but popularly cultivated throughout the world. A number of piperidine and pyrrolidine alkamides are known to occur in *P*. *nigrum*, the most important being piperine, known to possess a variety of biological properties like CNS stimulant, analgesic, antipyretic and antifeedant activities [7]. *S*. *grandiflora* (Family Fabaceae), commonly known as sesbania, is often planted in tropical countries for its edible flowers and pods. Leaves of *S*. *tora*, normally known as foetid cassia (Family Fabaceae), can be cooked as a vegetable whereas the seeds are used for treatment of ringworm and other skin diseases [8]. Leaves of *S*. *sesban*, another plant from Family Fabaceaue, are used in inflammatory rheumatic swelling and as an anthelmintic [9]. *M*. *oleifera* (Drumstick tree: Family Moringaceae), is used to combat malnutrition, especially among infants and nursing mothers. *M*. *oleifera*, contains a high level of vitamin A, vitamin C, potassium and iron [10]. *M*. *charantia* (Bitter gourd: Family Cucerbitaceae) is a vegetable with pantropical distribution that contains substances with antidiabetic properties such as charantin, vicine, and polypeptide-p, as well as other unspecific bioactive components such as antioxidants [11]. Fruit of *B*. *ramiflora* (Family Euphorbiaceae) is usually eaten fresh, stewed or made into wine, and the seeds are edible as well [12]. Several plants in this list have the various indications in Ayurveda medicine (an ancient traditional Indian system of medicine) [13].

The current study is designed to focus on the analysis and comparison of their melatonin contents using specific extraction and determination methods. The results of the study may support whether the soporific properties of these herbs could be attributed, in part, to their melatonin content. Our study is the first to analyze and compare the melatonin content of Thai herbs traditionally widely used as sleeping aids.

### Materials and methods

#### Plant materials

Mature leaves of *P*. *nigrum*, *S*. *sesban*, *S*. *glandiflora*, *M*. *oleifera*, *M*. *charantia*, *B*. *ramiflora* and *S*. *tora* were collected from Khon Kaen Province during August-September 2012. All plant samples were identified by comparing them with herbariums at the Forest Herbarium, Division of National Park, Wildlife and Plant Conservation, Ministry of Natural Resources and Environment, Bangkok, Thailand. Voucher specimens were kept at the Department of Pharmacognosy and Toxicology, Faculty of Pharmaceutical Sciences, Khon Kaen University, Bangkok, Thailand. The samples were dried in a hot air oven at 60°C for 6 hours, then ground by electronic hammer mill (Jacobson Universal Hammer Mill, Model L6DCE10, Jacobs Corporation, Indiana, USA) to produce powders. Dry weight of leaves was about 10% of fresh wet weight. Melatonin standard and other reagents were purchased from Sigma (St. Louis, MO, USA).

#### Melatonin extraction

Extraction and analysis was modified from the procedure previously described (10). Herbal powders (5g) were weighed in quintuplicate into 250 mL rotary evaporator bottles, sonicated with lab-grade 20 mL methanol (Lab-Scan Analytical Sciences, Poland) for 6 hours at room temperature in an ultrasonic water bath (Branson Model 3210, Branson Ultrasonics, Connecticut, USA), then volume reduced under vacuum (Eyela Rotary Evaporator, Tokyo Rikikiai Co. Ltd., Tokyo, Japan) at room temperature. The supernatants were filtered through Whatman No. 1 (Pittsburgh, PA, USA) using a Buchner funnel, then transferred to new bottles and dried under nitrogen gas. The residues were redissolved in 4 mL of 5% methanol–water solution and loaded onto 5 mL Sep-Pac C18 solid phase extraction (SPE) cartridges (Waters, Milford, MA, USA) after pre-conditioned with 2 mL methanol followed by 5 mL of organic free ultrapure water filtered with a 40 μm filter (Elga DV25 Purewater OptionQ system.. After washing with 10 mL of 5% methanol to remove interfering impurities, the retained melatonin was eluted at a low flow-rate using 5 mL of 80% methanol–water solution, then stored at 4°C until high-performance liquid chromatography (HPLC) or enzyme linked immunosorbent assay (ELISA) analysis.

#### Melatonin analysis by HPLC

Analysis of melatonin content in the herbal extracts was performed using a previously validated method as described [14]. Briefly, a SpectraSystems P1500 HPLC system with HiQ Sil C18 column (4.6 mm × 250 mm 5 μm) with FL2000 fluorescence detector (λ_ex_ = 284 nm, λ_em_ = 284) using mobile phase of 35% acetonitrile in pH 7.2 phosphate buffer and a the flow rate 1 mL/min and 25°C with injection loop volume of 20 μL. A standard melatonin calibration curve was used to quantify melatonin, with correlation of r^2^ = 0.9998 over a range of 7.2–180 ng/mL. The limit of detection (LOD) was 3 ng/mL (3.3 times the signal to noise ratio of 0.011mV) and limit of quantification (LOQ) was 10 ng/mL (10 times the signal to noise ratio). Intra-day precision was 3.72% and the inter-day precision was 5.21% at 25.2 ng/mL and 4.8% and 5.9% at 90 ng/mL respectively.

We chose HPLC with fluorescent detection (FD) as it have shown good specificity for indoleamines found in plants; their specific absorption and emission bands are based on their substitution and electron delocalization. Many studies have previously reported that melatonin was clearly separable from other potentially interfering indoleamines with HPLC-FD [15-20]. The extraction/purification step with solid phase extraction (SPE) further reduced the likelihood of interfering compounds. Previous studies [16,17] validated the HPLC-FD method as reliable for the quantitative analysis of melatonin and met Association of Official Agricultural Chemists (AOAC) requirements compared to LC-MS.

#### Melatonin measurement using ELISA

Non-extraction melatonin ELISA (RE54041, IBL International, Hamburg, Germany) was used as per the manufacturer’s protocol to verify the identity of melatonin in the samples. Samples of 100 μL were used directly without dilution, and 100 μL of standards and controls were also used. A plate reader (Anthos ELISA reader; Labtec Instruments, Sabzburg, Austria) with ADAP 1.6 software was used to measure optical density at 405 nm. Melatonin concentrations were analyzed after subtracting blank readings, and standard melatonin concentrations were fit to a 5-parameter logistics regression equation with an r^2^ = 0.9989. The specificity of this ELISA kit for melatonin is high, with cross reactivity stated by the manufacturer as 5-methoxytryptamine 2.5%, N-acetylserotonin 1.2%, 5-methoxytryptophol 1.2%, and serotonin <0.02%. Cross reactivity of other substances tested was less than 0.01%. The functional sensitivity was 1 pg/mL.

### Results and discussion

**Figure 2:**
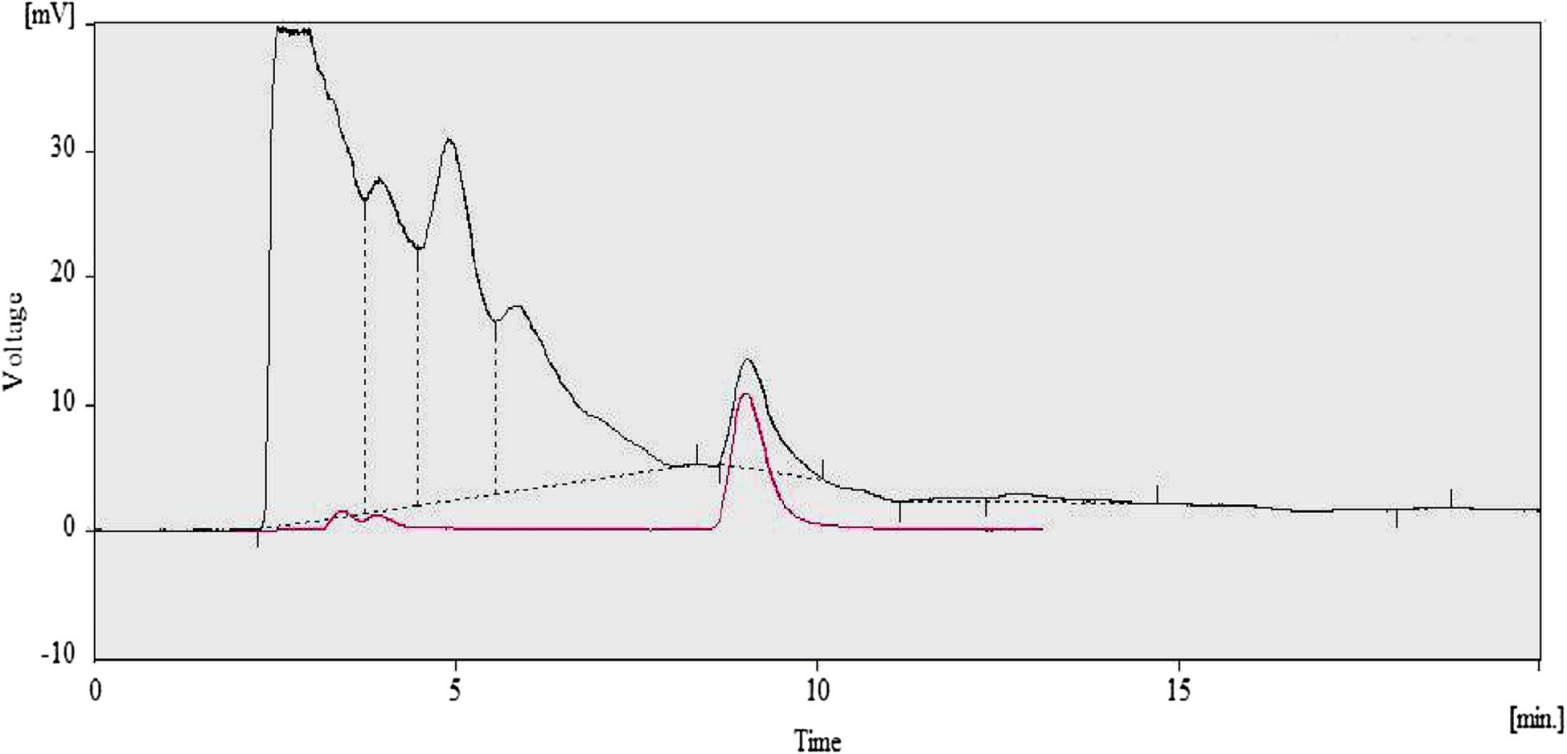
**Overlay of HPLC spectra of** ***Piper nigrum*** **extract (top) and standard melatonin (bottom).** Melatonin retention was 8.7 mins. Mobile phase 35% acetonitrile in pH 7.2 phosphate buffer, fluorescent detector λ_ex_ = 284 nm, λ_em_ = 284.

In this present study, melatonin was identified in extracts of seven herbs from a list of sleeping aids from Thai tradition medicine. The melatonin contents are listed in Table 1 from highest to lowest content. Melatonin peak was not detected in HPLC of the extract of *M*. *oleifera*. Overlay of HPLC spectra of *P. nigrum* extract and standard melatonin is shown in Figure 2.

**Table 1:** Melatonin content of herbs as determined by ELISA and HPLC

**Herb extract**	**ELISA melatonin ng/g dry weight**	**HPLC melatonin ng/g dry weight**
*Piper nigrum*	865 ± 243.4	1,092.7 ±34.1
*B*. *ramiflora*	76.7 ± 46.8	43.2 ±4.2
*S*. *glandiflora*	43.7 ± 7.3	26.3 ±2.6
*M*. *charantia*	N.D.	21.4 ±2.2
*S*. *tora*	N.D.	10.5 ±1.7
*S*. *sesban*	7.3 ± 2.8	8.7 ±1.3
*M*. *oleifera*	N.D.	N.D.

N.D. Below detection limit. ± = S.D.

Differences between the melatonin content found by ELISA and HPLC methods are not unexpected. Although immunoassay technique (ELISA) has been widely used for identification and quantification of melatonin in plants [19,21-23], and some studies have shown good correlation with HPLC [24-26], ELISA does have some cross-reactivity with structurally similar compounds, potentially causing an overestimation of melatonin content. In this study, ELISA was used to confirm the presence of the melatonin in samples in the absence of mass spectra (MS) data.

Melatonin was previously investigated in more than 100 Chinese medicinal herbs using solid phase extraction followed by HPLC and the highest content was found in Chantui (*Periostracum cicadae*) with a content of 3,771 ng/g, and 10 of the 64 herbs had levels higher than 1,000 ng/g. The study of Murch, Simmons, & Saxena, [27] found the melatonin content of feverfew varied between 1,370 and 2,450 ng/g of leaf depending on the preparation method.

A previous study has shown that tropical fruit or fruit juice containing melatonin, can contribute to systemic levels in the body [28]. For example, consuming the juice (500 mL) from 1 kg squeezed fresh orange containing 150 pg/g wet weight, and thereby provided 150 ng of melatonin, significantly increased serum melatonin during the day from baseline (from 40 to 151 pg/mL, P = 0.005). This is equivalent to normal nighttime levels endogenous levels (about 150 pg/mL) [29,30]. It would be expected, therefore, that herbal infusions of the recipe in this study, containing more than 1000 ng/g melatonin, would have sufficient bioavailability to contribute to melatonin blood levels, inducing sleep.

The high content of melatonin found in the *P*. *nigrum* suggests it may be very effective as a traditional medicine especially when used in combination with other herbs with sedative properties. This may explain the traditional formulation of a combination of seven herbs for sedative properties. Normally, leaves of *P*. *nigrum* are rarely consumed as a food unlike the other parts, such as the seed which is a very well known spice. The high melatonin content in the leaves is a promising result for future development of this overlooked part of *P*. *nigrum* as a health food supplement.

### Conclusions

A validated bioanalytical method was used for determination of melatonin in seven traditional Thai sleeping aid herbs using HPLC-fluorescence detection. Melatonin was extracted from other interfering compounds using solid phase extraction. One of the analyzed herbs, *P*. *nigrum* L showed an encouragingly high amount of melatonin (1092.7 ng/g of dry sample weight), within the range of the highest amount previously reported for dried herbs, while other herbs in the Thai herbal remedy contained modest amounts (8.7 - 43.2 ng/g of dry sample weight).

## Competing interests

We declare that we have no competing interests.

## Authors’ contributions

TP: Plant extraction, SPE purification, manuscript author. JJ: HPLC and ELISA analysis, and approval of the final version. AS: HPLC analysis of melatonin content. ST: Project advisor. All authors read and approved the final manuscript.
